# Computational Biology Methods and Their Application to the Comparative Genomics of Endocellular Symbiotic Bacteria of Insects

**DOI:** 10.1007/s12575-009-9004-1

**Published:** 2009-03-11

**Authors:** Jennifer Commins, Christina Toft, Mario A Fares

**Affiliations:** 1 Evolutionary Genetics and Bioinformatics Laboratory, Department of Genetics, Smurfit Institute of Genetics, Trinity College, University of Dublin, Dublin, Ireland

**Keywords:** Comparative Genomics, Orthologs search, BLAST, Functional Categories, Genomics Dynamics

## Abstract

Comparative genomics has become a real tantalizing challenge in the postgenomic era. This fact has been mostly magnified by the plethora of new genomes becoming available in a daily bases. The overwhelming list of new genomes to compare has pushed the field of bioinformatics and computational biology forward toward the design and development of methods capable of identifying patterns in a sea of swamping data noise. Despite many advances made in such endeavor, the ever-lasting annoying exceptions to the general patterns remain to pose difficulties in generalizing methods for comparative genomics. In this review, we discuss the different tools devised to undertake the challenge of comparative genomics and some of the exceptions that compromise the generality of such methods. We focus on endosymbiotic bacteria of insects because of their genomic dynamics peculiarities when compared to free-living organisms.

## 1. Genomes, Genomes, and More Genomes

The emergence of genome information has overwhelmed our efforts to analyze the unexpected amount of data generated during the last two decades. As an example, today (February, 2009), there are 438 complete microbial genomes and 17 in draft in the J. Craig Venter Institute, Comprehensive Microbial Resource website (URL: http://cmr.jcvi.org/tigr-scripts/CMR/CmrHomePage.cgi) considering that this is only a single resource we estimate that the number of completed genomes will be in the order of double that by the end of 2009 with a considerable percentage of these already published in the literature. Already the Entrez Genome project website controlled by National Center for Biotechnology Information (NCBI) reports that on February 3, 2009, 857 genomes are complete, 815 are in draft assembly, and 989 are in progress (http://www.ncbi.nlm.nih.gov/genomes/static/gpstat.html). The number of institutes worldwide with increasing sequencing capacities has been rising at an exponential rate and the first results of analyzing such data have solved old and long debated hypotheses and also have generated breakthrough ideas that have opened new avenues in all fields of genetics and evolutionary biology. However, our ability to cope technically with the amount of generated raw data has become seriously compromised, fueling many initiatives aimed at developing computational tools to analyze genomic and proteomic data. Many of these tools have been developed to perform comparative genomic analyses; each tool has had to face many of the complexities that biologically driven genome remodeling phenomena cause, such as genome duplication, rearrangement, and shrinkage. In this review, we first discuss the different technologies developed to perform genomic and proteomic analyses. We then focus on the importance of the developed tools to study biologically important phenomena such as genome duplication, the dynamics of genome rearrangement, and genome shrinkage that is associated with the intracellular life of bacteria.

## 2. Common Methods in Comparative Genomics

Comparative genomic methods are vast in number as well as function. A decision about the best way to do something is often a long and arduous task in this field, a task that has resulted in the design and reengineering of many of the tools that are available. To describe every method in this area of research would be next to impossible, and so, this text will provide a snapshot of what is available for many of the common tasks in comparative genomics. The logical place to start is of course the beginning—genome sequencing, assembly, and closing, then continuing to discuss the intricacies of comparative genomics.

While in the past comparative genomics has concentrated on sequencing single genomes and parts of genomes, current excitement lies with the sequencing of environmental communities. This field of research, entitled metagenomics is fast growing and the current hot topic. Its application is most utilized to characterize unculturable organisms (an estimated 99% of microbes cannot be cultivated in a laboratory environment [1]), but it has also made it possible to sequence genomes without the problems that are associated with cultures maintained in laboratories [2]. Metagenomics has transformed the uses of such organisms by allowing the focus to move from those that can be cloned in culture [3]. Depending on the source of the environmental sample to be subjected to environmental shotgun sequencing, a colossal variation in the number of identified species may result. Just looking at prokaryotes alone, as few as five species were identified in a community sequencing carried out on acid mine biofilm (Tyson et al. [4]), in contrast, as many as 3,000 species were sequenced from a soil sample taken in Minnesota, USA analyzed by Tringe et al. [5]. For a comprehensive review of this subject, *see *[6].

## 3. Sequencing

**Figure 1:**
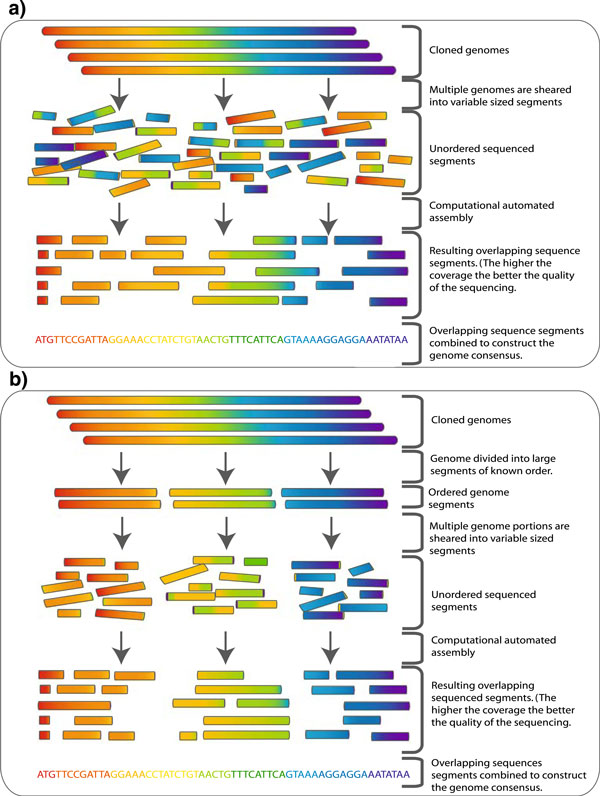
**a Whole genome shotgun sequencing: Genome is sheared into small approximately equal sized fragments which are subsequently small enough to be sequenced in both directions followed by cloning**. The cloned sequences (reads) are then fed to an assembler (illustrated in Figure 2). **b** To overcome some of the complexity of normal shotgun sequencing of large sequences such as genomes a hierarchical approach can be taken. The genome is broken into a series of large equal segments of known order which are then subject to shotgun sequencing. The assembly process here is simpler and less computationally expensive.

In the context of γ-proteobacteria, sequencing is commonly carried out using a shotgun approach. This technique is popular and is widely used in the generation of long sequences, such as those found in whole genomes. Briefly, this approach involves the sequencing of random small cloned fragments, known as reads, in both directions from the genome. This fragmented reading of the genome is carried out multiple times to provide good coverage and overlap within the sequencing. Having good quality overlap/coverage allows the reads to be assembled into their original order, thus reconstructing the genome (Figure 1a). Not surprisingly, reconstructing the genome from short overlapping reads is a nontrivial task and requires complex computational techniques to produce a quality result. This technique was first described by Sanger et al. [7] and has been refined and used as the basis of genome sequencing and assembly ever since. The method has been developed in two main directions: (1) a whole genome shotgun approach [7,8] and (2) a hierarchical shotgun approach [9].

As described above, the whole genome approach where the genome is fragmented into defined length reads is followed by assembly, using purely bioinformatic-based techniques. The second approach, which is more appropriate for larger genomes, utilizes an added step to reduce the computational requirement in assembling the final sequence (Figure 1b). Firstly, the genome is broken into larger fragments, which are in a known order; these fragments are then subsequently subjected to sequencing using the normal shotgun approach. This method requires less computational intervention in assembling the reads into the correct order. Information is already known about the order of each subset of reads and thus less error is incurred in the final assembly. Of course, there are disadvantages with each of these approaches. For instance, with the whole-genome approach, there is the uncertainty as to whether the assembly is correct due to the total reliance on bioinformatics tools to join and order the reads; in addition, coverage may be insufficient (i.e., overlap between the fragments). The second approach is time consuming and labor intensive due to the addition of the extra step at the beginning of the protocol [10]; this approach is also susceptible to incomplete coverage [11]. Further advances have been made since the advent of shotgun sequencing but the central concepts remain the same.

Technologies currently used in genome sequencing include high-throughput methods such as 454 [12], SOLid (Applied Biosciences), and Solexa [13]. These methods differ from older technologies in their throughput. Hundreds of thousands of DNA molecules at the same time are sequenced instead of a single DNA clones being processed [14]. The reads returned from each of these technologies are very short; thus, assembly is rather difficult. This disadvantage is offset by the fact that some much DNA is sequenced. The sequencing methodology of these approaches, in particular 454, is called pyrosequencing. This essentially is the sequencing of DNA utilizing the detection of enzymatic activity to identify the bases. This process is termed "sequencing-by-synthesis" [15]. Future developments will of course increase the length of reads produced by the technologies, as well as the accuracy of the programs with which the fragments are assembled.

Discussion in the past has provided some insight into the pitfalls of each method and perhaps aided in the decision making process [14,16,17]. One thing is certain, the higher the coverage the method is able to achieve, the higher the likelihood that the assembly tool will get the correct result and so that in itself should be one of the highest considerations in the decision making process.

## 4. Base Calling and Genome Assembly

After genome sequencing is complete, it then becomes necessary to reconstruct the sequence fragments into a meaningful order that will accurately reflect the original orientation and order of the gene and junk (noncoding regions and pseudogenes) content. The most common and popular manner in which this is achieved is through the Phred [18,19]–PHRAP [20]–CONSED [21] pipeline of tools (all of which originate from the University of Washington).

When assembling sequences from the myriad of reads that encompass a genome, several factors must be accounted for. Firstly, base-calling (the operation of determining the nucleotide base sequence from the chromatograph) must be completed with a minimum of erroneous interpretations of the chromatograph. The nucleotide sequence is determined for each read by the base-caller; the assembler then is utilized to piece the reads together into their original order, but must account for insertions, deletions, rearrangements, inversions, and sequence divergence in doing so. In particular, these events are important when assembling using a comparative method (i.e., using the scaffold of an existing genome to predict the locations of the fragments in the newly sequenced genome). No assembler (to date) proposes to handle all of these complications successfully but some do claim to be more capable than others under certain circumstances. For example, Pop et al. [22] reported that PHRAP [20] is more adept at creating long contigs (collection of contiguous pieces of DNA (reads)) than other available methods such as TIGR Assembler [23] or Celera Assembler (WGS-Assembler) [24]. This can be valuable and has been used in the past as an indication of the success of an assembler. More recently, it has been reported that a reduction in the length of contigs across the assembly is an acceptable outcome if the error rate is reduced [25]. Probably the most widely used base-calling algorithm is implemented in Phred [18,19]. Others include GeneObject [26] and Life-Trace [27].

PHRAP has been widely adopted as an integral component of assembly pipelines such as implemented by Havlak et al. [28] in the Atlas Genome Assembly System and Mullikin and Ning [29] in the Phusion Assembler. It is considered the standard way in which to assemble smaller genomes with larger genomes relying on more complex algorithms provided by programs such as the WGS-Assembler.

**Figure 2:**
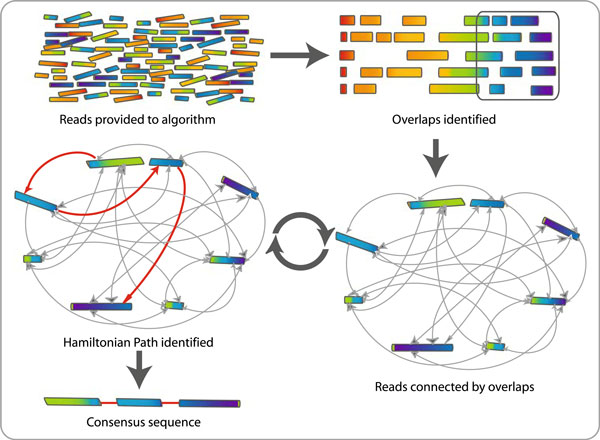
**Overlap–layout–consensus genome assembly algorithm: Reads are provided to the algorithm**. Overlapping regions are identified. Each read is graphed as a node and the overlaps are represented as edges joining the two nodes involved. The algorithm determines the best path through the graph (Hamiltonian path). Redundant information (i.e., unused nodes and edges) is discarded. This process is carried out multiple times and resulting sequences are combined to give the final consensus sequence that represents the genome.

Traditionally, assembly algorithms employ a method known as "overlap–layout–consensus" [30] (Figure 2). Initially, the reads are compared to one another to identify overlapping regions using a strategy known as hashing to minimize the time required to complete the computation [31]. When the potentially overlapping reads are positioned, a computationally intensive multiple sequence alignment is carried out to produce a consensus sequence. This consensus sequence is a draft of the genome and requires further computational and manual intervention to reach completion. In some genome assembly pipelines, a further step is introduced, in which information from sequencing in both directions of each fragment is utilized to reconstruct contigs into larger sections. These sections combine to create a scaffold, minimizing the amount of potential misassemble that may be introduced. Newer methods such as described by Pop et al. [31] eliminate the overlap identification step in favor of moving directly to the creation of the multiple sequence alignment, thus reducing the amount of time required to construct a draft assembly considerably. These methods have been entitled "alignment layout consensus" and are implemented in the AMOS Comparative Assembler (AMOS-Cmp). AMOS takes advantage of already available programs in its creation of multiple sequence alignments and scaffolds. Bambus [32] is designed to create scaffolds based on the discrete reads resulting from the shearing process of the shotgun technique. It aids in the resolution of the placement and direction of the reads using the mate-pair information produced by sequencing each read in both directions (a process known as double-ended shotgun sequencing). Using this scaffolding approach interleaved with other assembly techniques gives an elevated probability of producing a high quality complete genome.

There is no up-to-date objective comparison of genome assemblers available that takes the consistent development being carried out on each project into account. Comparisons carried out by groups such as Huang and Madan [33] and Chen and Skiena [34] are works that seek to validate recently released methods. Chen and Skiena [34] come closest to an objective comparison in their rigorous testing of their own creation, STROLL, and latest versions (at the time) of PHRAP by Green [20] and the TIGR Assembler by Sutton et al. [23]. In their evaluation of the programs, they reported that PHRAP was consistently more accurate in producing the correct assembly and had the lowest error rates of the group. STROLL produced similar results to PHRAP while TIGR Assembler produced a considerably more erroneous resultant assembly. The TIGR Assembler produced significantly more and smaller contigs, a higher proportion of gaps remaining unclosed and aside from the result, the process of running the TIGR Assembler on the read data used took approximately five times longer to complete than either of the other two programs evaluated.

In the race to publish the Human genome in the early 2000s, the Celera Whole Genome Assembler was engineered to accommodate large genomes. Its first use was described by [24] in the paper reporting the completion of the *Drosophila* genome (Myers et al.). This was enhanced and used later in the initial assembly of the Human genome [35] and the publication of the whole human genome assembly [36] in addition to the mouse [37], dog [38], and mosquito [39] genomes. While Celera is a private corporation, it has released the Celera Assembler as open source software for free usage.

In early 2007, a new assembly algorithm was described by Sommer et al. [25]. It is a streamlined approach aimed at providing a simple, faster, and more efficient means of assembling fragmented sequences. Minimus [25] performs its best on small assembly jobs such as small genomes, genes, and bacterial artificial chromosome clones [40]. It has also been assessed with respect to assembling larger sets of fragmented DNA such as those found in bacterial genomes and has been found to produce fewer assembly errors than PHRAP. The cost of this reduction in error rate is that the number of contigs is greater and consequently, the size of the contigs is smaller, resulting in a more fragmented assembly [25]. In addition, all test assemblies produced by Minimus were completed in approximately half the time that PHRAP used. It remains to be seen whether this new assembler will work its way into common use in assembly systems such as Phusion and Atlas, but it is unlikely to remain at an advantage for long as the development and advancements of new and reworked as assemblers is swift and continuous. It has been suggested that it is beneficial for more than one method to be used, so that the exclusive advantages of each method may be exploited [33]. This strategy may well of course be more time consuming but if this time is affordable, it should be implemented.

## 5. Annotating the Genome

Distilling information from the assembled genome is the next obvious step in the process of building biological understanding of each newly sequenced individual or species. Genome annotation has three main levels—nucleotide-level annotation, protein-level annotation, and process-level annotation. The DNA level annotation process itself has several procedures associated with it. The first procedure is called Mapping, which is the process of identifying known genes, markers, and landmarks within the genome. This is usually carried out using sequence similarity searching programs such as BLAST [41]. Secondly, Gene Finding as the name indicated involves the prediction of gene locations within the genome. Within the genes, the location of introns and exons are sought out in an effort to characterize the DNA into coding and junk categories. This is not a trivial process and often result in very poor sensitivity and specificity, in particular, results are poor when the signal-to-noise ratio is low, i.e., the amount of noncoding DNA is high (for a more elaborate review and comparison of gene prediction algorithms, *see *[42]).

Due to the extraordinary numbers of genes and sequences that have already been characterized in one species or another, a lot of the effort required to identify genes is cut out. Also to be identified are noncoding regions including, for example, tRNAs and rRNAs. These are mostly characterized by means of once again similarity searches and by using programs such as tRNAScan-SE [43]. Other regions that must be discovered are regulatory regions, such as transcription factor binding sites, the topic of which is covered in detail in a review paper [44]. In brief, methods have been developed to identify these regions by looking for patterns that occur more often that would be expected by chance; often this strategy is carried out in conjunction with similarity searching techniques.

At the protein-level annotation step, characterization is carried out. Genes are named and assigned functions mostly by means of comparison to already annotated genomes. Often this results in the categorization of many proteins into "unknown function" or "hypothetical protein" categories until experimentation provide light on the purpose of the gene at hand.

The final level of annotation is Process. Here, the biological processes affected by the gene are identified. Process categories usually include cell cycle, cell death, immune response, metabolism, etc. to name but a few. Once again, the processes affected are usually determined via comparison with the information that is already available. It is useful here to note the existence of a few well-established databases that have devised naming conventions and controlled vocabulary for the description of new genes. Probably, the most commonly utilized of these are Panther [45-48] and GO [49,50]. Both of these are freely available for use via the World Wide Web and are widely accepted adhered to by the genome analysis community.

Much work has been done in the development of quicker and more reliable ways of dealing with and identifying the protein coding regions of a genome at the same time noncoding regions while not completely neglected have been lesser studied of the two. Neither the detection of coding or noncoding regions is easy nor is the development of reliable and robust methods nearing a plateau. Constant progress is being made in these field; thus, the literature should be watched closely in order to be up to date with the current best practices in annotation.

## 6. Closing the Genome

Closing and completing a genome-sequencing project has proved to be an important step in ensuring the accuracy and reliability of the output into public databases. While the release of draft sequences is very useful, they are notoriously erroneous—in sequence and assembly [17]. Error rates for draft sequencing have been reported to be 1 in 1,000–2,000 base pairs [51], in contrast to the rates of 1 in 10,000 reported by Selkov et al. [51] and 1 in 100,000 reported by Fleischmann [52] for whole genome sequencing. The typical errors found in draft sequences are sequencing errors, sequence misassembles, and the inclusion on contaminant sequences from foreign DNA as bona fide reads [17]. Finding the source of such problems is difficult and time consuming and is often carried out manually. The most important factor taken into account here is the economic tradeoff and whether it is worth the compromise. For example, are there enough financial resources to allow for the whole genome sequencing to be brought to a close? It is important to realize that the quality of the sequencing or lack thereof will propagate forward into whatever analysis is carried out using the DNA sequence. Negative effects will be evident in all downstream analysis; everything from annotation and gene recognition to subsequent identification of homologs, gene families, and phylogenetics relationships will be affected.

While the discussed methods of sequence assembly are thorough and have relatively low error rates, they are not capable of producing a completely reconstructed genome sequence without manual intervention and some potential resequencing. What the methods do produce is a draft sequence that would normally cover approximately 99% of the genome under reconstruction [17]. This draft stage of assembly can be reached within a short number of days. In contrast, the process of closing the assembly out may potentially require months to complete and in some instances may take years. For example, the draft human genome was published in 2001, 4 years ahead of the predicted date of availability (2005). The complete whole genome was, however, not finished until 2003 and subsequently published in 2004 [36]. The time and consequently the monetary cost incurred is a sacrifice that those in the area of comparative genomics are willing to make, as the quality provided by a closed genome is well worth the wait. Moreover, while useful in their own right, draft assemblies are constantly changing and potentially erroneous.

To meet the need for high quality complete genome sequences, several strategies have been developed at facilities such as TIGR, Washington University and Sanger. In some cases, a certain amount of error checking is carried out in conjunction with assembly. Programs such as EULER [53] and Arachne [54] are examples of assembly systems that include error correction components. Other approaches include the use of correction algorithms a posteriori to the assembly process. Examples of this type of program are Autofinish (of the wider package—CONSED) [55], MisEd [56], and ReDit [57]. Autofinish, one of the most popular computer programs, is used in many genome sequencing centers, such as The Genome Center at the University of Washington, the Berkeley Drosophila Genome Project at Lawrence Berkeley National Laboratory, and the Lita Annenberg Hazen Genome Center at Cold Spring Harbor Laboratory among others [55]. The product of the program must be manually inspected to ensure the quality and accuracy, but the amount of human intervention in this program is significantly reduced. In projects that had sequence coverage as low as four and five times, the human time required to close the project was reduced by more than 51% and 83%, respectively [55]. As the sequence coverage increased up to 14 times, the difference diminished, but consistently less human effort was required when Autofinish was utilized.

The finishing techniques that are employed in programs such as Autofinish reflect what a human finisher does in identifying problem areas in the assembly that has been produced. They go on to propose possible means of resolving the issues, indicating regions to be resequenced and potential reads to aid in closing any gaps that are present. Due to the nature of the problems that are found in draft genome sequences, the process of finishing is an iterative process that can require many cycles through a workflow to resolve all issues; frequently, it is necessary for a human finisher to get involved toward the end to complete the process. This intervention must be as efficient as possible and many graphical viewers and editors are available for this purpose. Examples of manual finishing software are components of the aforementioned CONSED: sequence finishing tool [21] and ReDit: shotgun assembly finishing aid [57], also others include BaCCardI: validate and assist in finishing [58] and DNPTrapper: analysis of complex regions and finishing tool [59]. Each of these software programs aim to make the editing process as user friendly as possible while offering the best possible combinations of editing capabilities.

## 7. Comparative Genomics: Solving the Puzzle

Comparative genomics is one of the most promising areas that logically follows the success in improving genome sequencing. More and more comparative genomics programs are being demanded to identify protein-coding genome regions, placement of regulatory elements, and the main evolutionary dynamics affecting the complexity of genome organization. Despite its apparent simplicity, such comparative methods have to face many technical as well as theoretical problems. One of the most important problems is aligning whole genomes and visualizing such alignments in a comprehensive and comprehensible way. This problem in sequence alignment leads to other genomic problems such as the finding of orthologs between genomes. The magnitude of this problem becomes increasingly magnified when the comparison is held between genomes with different population dynamics and hence different mutational rates, as we will explain below.

### 7.1. The First Hurdle—How to Determine the Homologs (Orthologs and/or Paralogs)?

Identification of homologous genes relies on the appropriate definition of a homolog. The most widely accepted definition is that homologous genes share a common ancestry. This definition, however, is not precise as to the nature of this common ancestry and comprises two types of homologs (as described by Fitch [60] and Fitch and Margoliach [61]): orthologs (common species ancestry caused by speciation event in such away that the homolog genes are in different species) and paralogs (common gene ancestry caused by a gene duplication event and, as a consequence, the homologous genes are present in the same species).

Irrespective of the nature of the ancestry considered, homologs are usually identified on the basis of sequence similarity. So the higher the similarity, the more likely it is that the sequences have derived form a common ancestor. One of the first and the most commonly used software to detect the degree of similarity between sequences is BLAST [62] and the newer version PSI-BLAST [63]. BLAST uses predefined scoring matrices in comparison to position-specific scoring matrices derived from the scoring hits in the initial search in PSI-BLAST. The two programs yield information about the score for the comparisons and their likelihood, called the *e*-value. Sequences with the highest scores and therefore with the lowest *e*-values are considered to be the closest relatives in the searched database. The assumption underlying this software is that the phylogenetic relationship between any two sequences and their degree of similarity are positively correlated. This, however, leads to another theoretical problem: how to determine if a sequence is more similar to a different particular sequence than it is to another. Unfortunately, setting a statistical cutoff value to determine when two sequences are significantly similar is rather difficult and problematic when determining a set of possible homologs. The lower the cut off, the larger the number of false negatives. On the other hand, the higher the cut off, the larger the number of false positives. As an additional drawback, the sequences with the highest score and lowest *e*-value are not always more closely related to each other than those identified as hits with a lower score [64].

In the BLAST searches for homologs, many types of relationships between the homologs can be investigated, including hits of many-to-many, one-to-many, or very strict one-to-one relationships. The first two are a result of duplication events after speciation. A very effective way to identify one-to-one relationship is by performing the generally called reciprocal best BLAST hits [65,66]. This method is based on the assumption that genes that are each other's best hits when performing a BLAST search are more likely to be orthologs compared to ones that are not. The reason for this is that although gene A in genome 1 may be the best match for gene B in genome 2, this match may be worse than gene B in genome 2 with gene C in genome 1. This approach is again limited by the problem of the assumption that best hits ensure orthology, which might not be the case when a particular gene underwent a recent duplication in a particular lineage. The consequence of this is that when a gene finds a paralog as top BLAST hit instead of its ortholog, both the gene and its paralog are excluded from downstream analyses [67]. These limitations in the BLAST searches have fuelled the development of other ways to identify putative orthologs over the last few years. One of such methods uses the sequence distances instead of similarities to identify orthologs and uses the reciprocal smallest distance algorithm [67]. It uses global sequence alignment and maximum likelihood to estimate the evolutionary distances between genes to detect orthologous genes. This approach have also been used to determine orthologs in databases like Roundup [68]. Another simple approach that has contributed significantly to the reduction in the number of false positive results when conducting BLAST searches is PSI-BLAST [69].

Homology may also be ascertained by means of phylogenetic methods such as BranchClust by [70]. This type of method is capable of determining homology distinguish it from paralogy. BranchClust utilizes similarity searching during the execution of its algorithm but obviously does not rely solely on it. Hits within a certain threshold are used rather than the best hit in order to include paralogs and orthologs. These results are then grouped into what Poptsova and Gogarten has termed superfamilies. These sequences are aligned and phylogenetic trees are constructed. The step of phylogenetic inference is then followed by a complex algorithm that is described fully in the application's article [70]. The outcome of using this method over more traditional one is that BranchClust is reported to outperform similarity search methods due to its lower false negative rate than the reciprocal best blast hit method.

Irrespective of the method used to identify homologs, visualizing results is a common way to inspect and yield the first insights into trends and patterns when looking at genome evolutionary dynamics. This fact has inspired the creation of software for comparative genomics with graphical solutions to assist in the interpretation of the results. These solutions provide user-friendly environments in which navigation along alignments, etc. is easy and reliable. The question remains, however, whether visualization tools can solve the puzzle of genome rearrangements. An argument against the use of techniques such as this is that the process will not be repeatable or statistically sound. Undoubtedly, insights will be yielded but all sure perceived trends should be investigated in a more analytically robust manner.

### 7.2. Pairwise Genome Comparisons

**Figure 3:**
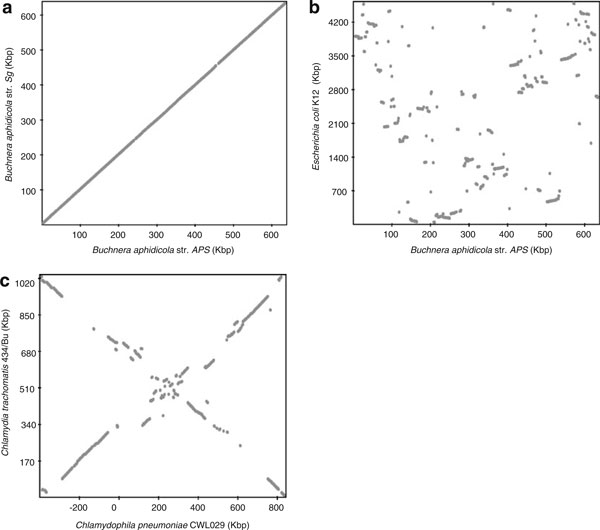
**Genome rearrangements plots comparing two genomes**. Genome plots can provide information on the kind of rearrangements undergone. These plots represent the location of each gene in one axis for one of the genomes against the location of the found ortholog in the other axis for the second genome. **a** Comparative genomic plot when comparing two genomes showing no lineage-specific genome rearrangements. In this case, the plot was produced for the comparison of two primary symbiotic bacteria of insects (*B. aphidicola* strain *A. pisum* versus *B. aphidicola* strain *Schizaphis graminum*). Since no rearrangements have occurred in any of the two genomes, the comparison yields a straight diagonal line. **b** Comparative genomic plot for two genomes showing lineage-specific genome rearrangements. In this case, the plot was comparing the genome of other patterns that can be observed and are *x*-like patterns **b** (in this case, *B. aphidicola*, *A. pisum*, and *E. coli* k12) where the rearrangements have occurred over the replication axis *E. coli* K12 to the genome of *B. aphidicola* strain *A. pisum*. **c** This is the comparison between *Chlamydophila pneumoniae* CWL029 and *Chlamydia trachomatis* 434/Bu that show an even better example of rearrangements that have occurred over the replication axis (this example have also been shown in [102]). As shown, many rearrangements including inversions and translocations have occurred, and consequently, the orthologs are not located in the major diagonal of the plot but rather show an *X*-shape distribution. This is expected if an inversion has taken place near the centromer of the chromosome.

Many groups have devoted a substantial amount of their resources to the development of tools aimed at comparing two genomes and have validated such tools by comparing circular prokaryotic genomes. Some visualization software tools have specialized in performing direct comparisons of synteny information through scatter plots of pairwise genome comparisons. For example, software such as DAGchainer [71], GeneOrder [72], GenePlot from NCBI [73], Genome v/s Genome Protein Hits Scatter Plot from The Comprehensive Microbial Resource (CMR) [74], and GenomePlot from PLATCOM [75] achieve this by presenting a plot where one axis represents the positions of the genes within one of the genomes while the other represents the genes for the other genome (Figure 3). The scatter plot then represents homologous genes for both genomes determined by either total hits or best BLAST hit. Perfectly syntenic genes between the two genomes would therefore represent a linear relationship between the two axes (Figure 3a) whereas alternative arrangements of the scattered dots may indicate that genome rearrangements have taken place in one of the genomes (Figure 3b). As an alternative to these visual representations, other programs such as GRAST mark the hits between the two genomes and represent them in a circular way [76]. Finally, other programs such as ACGT [77], GOAL from BROP [78], BugView [79], and GenomeComp [80] have contributed to the field of comparative genomics by linearly representing rearrangements or syntenic information by linking homologous regions between the compared genomes using lines. The advantage of programs such as these is that in addition to yielding information about genome rearrangements, they can also spot conserved and nonconserved regions between the two genomes in much greater detail than other programs.

Aside from the syntenic analyses using visualization tools, other programs have been developed to search for other types of information in comparative genomics. For example, GC Comparison Graph from The CMR [74] compares the GC content between two genomes by placing orthologs in the axis according to their GC content, highlighting GC compositional shifts at the genome level between two genomes. Although useful in their content, these programs are subject to several drawbacks from the pragmatic point of view among which the most important is the impossibility to perform multiple genome comparisons and hence to establish the ancestry of genome rearrangement dynamics.

### 7.3. Multiple Genome Comparisons

As the number of genomes increased over the last decade, the demand for an understanding of the dynamics of genome evolution also increased. Dealing with the complexity of multiple genomes comparisons has been halted by the unparalleled development of appropriate software tools. Nowadays, several software tools have been developed. An example of a multiple genome comparison tool is GenColors from Jena Prokaryotic Genome Viewer (JPGV) [81]. This program allows the user to display a number of features on the genome, like CDS, RNA genes, tRNA genes, rRNA genes, Mics RNA, GC contents, GC skew Keto excess, etc. This database also represents genomes in either a circular diagram or in a linear plot. Although several genomes can be examined at the same time using this tool, these are human observations of the genomes rather than real phylogenetic studies of the genome properties. JPGV allows multiple genome comparisons by determining a core gene set of two or more genomes defined by the set of best-bidirectional hits for all possible pairs of genes. Other methods of the JPGV are implemented to perform pair wise comparisons only.

In addition, there are computational tools that compare multicircular prokaryotic genomes and present their similarities in a circular diagram. Some of these tools perform these comparisons in addition to the BLAST searches and the CGView server is an example of that [82]. Others also display information about the percentage of GC for each one of the genomes, such is the case of GenomeViz [83].

To gain more information about genome rearrangements and inversions, there has been a great effort in developing tools that perform linear comparisons between genomes. The way these tools compare genomes is by performing genomes alignments where possible and then by conducting multiple genome comparisons. There are many different multiple genome alignments algorithms. The first type is based on defining a reference genome and performing alignments taking into account that reference genome. This type of alignment algorithm is implemented in a program called Vista [84]. The second approach is that where an iterative pairwise alignment is performed under the control of a guide tree. The tree defines the order in which the genomes should be added to the alignment. The third type of algorithms determines anchors present in all genomes and then proceeds to align them. Once aligned, the last step is to close the gaps between the anchors by aligning the substrings between them. Examples of programs implementing this type of algorithm are MGA [85], M-GCAT [86], and Mauve [87], with each of them having their own algorithm for identifying the anchors and performing the alignment of the interanchor regions afterward.

There are other tools that allow the user to do other things in addition to the alignment of genomes. For example, MANTIS [88] is a phylogenetic-group specific (metazoan phylogeny) tool that analyzes the patterns of gene gains and losses at specific branches of the phylogeny. Then, the program infers the gene content of the ancestral genome to the clade and identifies over- or underrepresentation of certain processes among the class of gene gains or losses.

Despite all these effort in developing more robust and accurate methods to perform comparative genomic studies, several biological phenomena pose difficulties in identifying the real genome dynamic processes in organisms. For example, genome duplication, genome shrinkage in intracellular symbiotic bacteria, and lateral gene transfer may well hide the real genome rearrangement processes undergone in particular genomes. To illustrate the importance of the biology of the organismal biology to understand genome dynamics, we will focus the rest of the review on intracellular bacterial genomes.

## 8. Comparative Genomics of Intracellular Bacteria

Intracellular bacteria are a special group of organisms that have been able to adapt to intracellular life, establishing either a symbiotic or pathogenic relationship with the host. Because many of the genes that were important for the free lifestyle are no longer needed by these bacteria, they underwent nonfunctionalization followed by disintegration [89]. This process has been enhanced by the fact that the host provides these bacteria by some of their needed components and by a chemically stable rich environment. Genome shrinkage is therefore a fact in most if not all the strict intracellular bacteria and this process has been mostly accompanied by genome rearrangements and fast evolutionary rates of proteins. Because of these intracellular associated genomic and evolutionary events, comparative genomics including identification of orthologs, paralogs, synteny analyses, and others pose great challenge in the comparison with free-living bacteria and require including biological information in the comparative genomics analyses to increase the accuracy of the results.

In the case of the symbiotic relationships, the difficulty of comparative genomics acquires another dimension and complexity specifically associated to the mutational dynamics of these organisms. There are two main groups of symbiotic bacteria: the facultative and the obligated. When the association is facultative, it implies that the survival of each partner can be possible without the other under special environmental conditions. This is for example seen between the pea aphid *Acyrthosiphon pisum* and the facultative endosymbionts *Hamiltonella defensa* that acts as a protector of the aphid against parasitism by the solitary endoparasitoids *Aphidium ervi* and *Aphidius eadyi *[90-92]. The other case, obligated, is when the relationship between the two organisms becomes so close that the host's relative biological fitness would become seriously compromised if deprived of the symbiont. This is the case of the symbiotic relationship between the bacterium *Buchnera aphidicola* sp. and the aphid insect [93] and it is an example where the host (the aphid) has evolved specialized cells to house its endosymbionts (so called bacteriocytes) [94]. This relationship is one of the best characterized in the literature so the last following part of this review will focus on endosymbionts contained in bacteriocyte and the challenges that their mutational dynamics impose in the comparative genomics of bacteria.

### 8.1. Genome Evolution of Intracellular Bacteria

The clonal vertical transmission of small populations in many intracellular symbiotic bacteria and pathogens to the next host generations imposes a strong bottleneck on the effective population size of these bacteria. This results in relaxed selective constraints in the symbiotic genomes and their channeling into a dynamic of neutral fixation of slightly deleterious mutations and irreversible increase in the endosymbiont genome mutational load (a phenomenon named Muller's ratchet [95]). However, these bacteria are also subjected to selection imposed mostly over their insect hosts. Because of their clonal transmission and their confinement to the interior of bacteriocytes symbiotic bacteria have little or no opportunity for recombination and hence have no alternative means for the removal of these slightly deleterious mutations.

Is there a minimum set of genes necessary for the maintenance of intracellular life? Numerous scientists have addressed this question and many have been attempting to answer it through the study of the smallest endosymbiotic genome [96]. Comparative genomics studies in a large number of organisms have shown that the minimal gene content will depend on the environmental conditions the organism lives under [97,98].

The process of gene loss in intracellular organisms has an important effect on rewiring the functional relationships among genes. This would lead to different organisms containing different genes performing the same essential functions in the cell. So when looking at gene content of intracellular bacteria, we should talk about the functional group of genes instead of individual genes [99].

### 8.2. Difficulties with Comparative Genomics of Bacteriocyte-Housed Insect Endosymbionts

Comparison of bacterial genomes may provide clues about the main genome rearrangement dynamics supporting different lifestyles, for example, comparative genomics of intracellular symbiotic bacteria and their closest free-living relatives. Performing comparative genomics on bacteria that are in an intermediate stage between free-living and host specific symbiosis (primary endosymbionts) with each of their groups could shed some light on the establishment of symbiosis itself. These bacteria are the ones we refer to as secondary endosymbionts—they are distinguishable from primary symbionts by their larger genomes and the fact that they are not living under the protection of the bacteriocytes provided by their hosts.

As a consequence of Muller's ratchet in intracellular bacteria in combination with mutational bias, their genomes present a higher AT content than observed in their free-living relatives [100]. This results in programs like BLAST having increased difficulty in determining homologs—especially between the intracellular bacteria and their free-living relatives.

The difficulty of doing comparative genomics with intracellular bacteria is that few to none of the software programs have been designed to deal with any of the theoretical problems seen in these organisms. Most software and methods have been directed toward the broad stream of the comparison of genomes with similar sizes and belonging to bacteria with minor differences regarding their lifestyle or environmental conditions. The challenge, however, resides on identifying important genomic dynamics that occurred during the transition between two lifestyles and hence between potentially different biological systems.

One of the biggest problems with the comparative genomics of endosymbiontic and pathogenic bacteria to their closest free-living relative bacteria is the different evolutionary force under which they evolve. Because the population sizes of endocellular symbiotic bacteria undergo strong bottleneck during the intergenerational transmissions, many of the stochastically produced amino acid mutations are fixed by genetic drift despite their slight deleterious effects. This implies that the mean mutational load in the endocellular bacteria will dramatically increase posing serious difficulties to find their orthologs in free-living bacteria. Comparing endosymbionts with each other can yield valuable information about endosymbiosis but it is crucial to compare the endosymbionts to free-living bacteria to be able to investigate the transition from free-living to intracellular lifestyle and predict the shift in evolutionary forces. Novel methods are hence required to account for the biological and population genetics differences of the organisms whose genomes are being compared.

### 8.3. Databases and Methods for the Analysis of Endosymbionts

BuchneraBASE [101] is a database that contains information on *Buchnera* sp. APS. This database is the only of its kinds, to our knowledge, devoted completely to a primary symbiont. It does not offer any direct comparative genome tool for the user like many other databases but it contains some data obtained from comparison between symbiotic gamma-proteobacteria and an in silico model of *Escherichia coli*. This database was built as to integrate new sequenced genomes from symbiotic bacteria as they became available. It performs comparisons between different genomes using the information of gene orthology. The database also has a summary page that shows two user-interactive tables. The first table represents the number of genes in each of the genomes that are in a certain category, i.e., total number of complete genes, total number of pseudogenes, genes with an *E. coli* ortholog shared with the endosymbiont of *Wigglesworthia glossinidia* or not shared with *Wigglesworthia*, etc. The second table can be used to browse through each of the functional classifications for each of the symbionts stored in the database.

To our knowledge, there is only one program, GRAST [76], that has been developed with the sole purpose to investigate the evolutionary dynamics of endosymbionts. It performs a pairwise comparison between a free-living (reference genome) genome and an endosymbiotic genome and allows the user to choose between different outputs options, providing valuable insights regarding the change in genome dynamics in comparison to their free-living relatives. The outputs range from generation of genome plots with orthologous and nonorthologous genes' sets are plotted in the two genomes being compared to plots with the analysis of the distribution of genome rearrangements or dynamics in one of the genomes (Figure 3). Among other types of information, the program yields information about conserved regions between the two genomes, distribution of percentage of differences in the number of genes present in the different functional categories between the two genomes being compared and deviations from the expected percentage of orthologs between the genomes, and information about intergenic regions according to their position/rearrangement in the two genomes.

A brief look at the genome sizes of bacteria would suffice to realize about the incredible diversity of the genomic dynamic events that have been happening throughout evolution. These events are key to understand the different evolutionary processes shaping organismal organization. Intracellular organisms perform a minority of this diversity but they represent extreme cases where most of the genomic dynamics become dramatically manifested. New methods should therefore be developed to perform in-depth comparative genomic analyses of these bacteria to infer important shifts in the evolution of genomes.

The genomic era has exploded and generated new research avenues that go beyond all expectations. A plethora of novel ways of designing experiments and computational tools has been fuelled by the information generated from the first comparative genomics analyses. The challenge that remains is to design new comprehensive and accurate bioinformatics tools capable of counterbalancing our limitations to analyze the overwhelming amount of genomic data generated.
